# Analysis on International Competitiveness of Service Trade in the Guangdong–Hong Kong–Macao Greater Bay Area Based on Using the Entropy and Gray Correlation Methods

**DOI:** 10.3390/e23101253

**Published:** 2021-09-26

**Authors:** Xianhang Xu, Mohd Anuar Arshad, Arshad Mahmood

**Affiliations:** 1 School of Innovation and Entrepreneurship, Chongqing Institute of Engineering, Chongqing 400056, China; xuxianhang@cqie.edu.cn; 2 School of Management, Universiti Sains Malaysia, Gelugor 11800, Penang, Malaysia; 3 School of Business and Economics, University of Wuppertal, 42119 Wuppertal, Germany; arshad@usm.my

**Keywords:** Guangdong–Hong Kong–Macao Greater Bay Area, service trade, international competitiveness, entropy and gray correlation, industrial synergy

## Abstract

Based on the analysis and measurement of the overall situation, import and export structure and international competitiveness of the various sectors of service trade in the Guangdong–Hong Kong–Macao Greater Bay Area, with the help of MATLAB and Gray System Modeling software, the synergy degree model was established to quantitatively analyze the synergy level of service trade in the Greater Bay Area with the help of grey correlation analysis method and entropy weight method. The results show that the overall development trend of service trade in the Guangdong–Hong Kong–Macao Greater Bay Area is good. The service trade industries in different regions are highly complementary and have a high degree of correlation. The potential for the coordinated development of internal service trade is excellent, and the overall situation of service trade in the Greater Bay Area is in a stage of transition from a moderate level of synergy to a high level of synergy. The Greater Bay Area can achieve industrial synergy by accelerating industrial integration and green transformation, establishing a coordinated development mechanism, sharing market platform, strengthening personnel security, and further enhancing the international competitiveness of service trade. The established model better reflects the current coordination of service trade in the Guangdong–Hong Kong–Macao Greater Bay Area and has good applicability. In the future, more economic, technological, geographic, and policy data and information can be comprehensively used to study the spatial pattern, evolution rules, and mechanisms of coordinated development in the broader area.

## 1. Introduction

With the deepening of globalization, the world economy is gradually transforming into a service economy. As a dynamic factor of international trade, the service trade is characterized by less pollution, low consumption, high value, and sustainable development. It is a typical smart and low-carbon industry and is regarded as an effective way to promote economic transformation and development by countries all over the world. It has become a powerful driving force for global trade and economic growth. China’s economy is shifting from a stage of rapid growth to a stage of high-quality development and the new round of opening-up, focus on the service industry continues to deepen. Service trade will boost China’s industrial transformation and high-quality economic growth. The Guangdong–Hong Kong–Macao Greater Bay Area includes Hong Kong, Macau and Guangzhou, Shenzhen, Zhuhai, Foshan, Huizhou, Dongguan, Zhongshan, Jiangmen, and Zhaoqing. As the fourth pole of the global bay area after San Francisco Bay, New York Bay, and Tokyo Bay, it is gradually taking shape. Most of China’s Fortune 500 companies, unicorn companies, high-tech companies, and R&D resources are concentrated in the Guangdong–Hong Kong–Macao Greater Bay Area [1]. The Guangdong–Hong Kong–Macao Greater Bay Area is expected to become a world-class city cluster and an important hub for global innovation networks. The Guangdong–Hong Kong–Macao Greater Bay Area is not only the vanguard of China’s “second opening”, but it also plays a role in leading regional innovation and driving global economic growth. According to the development experience of the San Francisco, New York, and Tokyo Bay Areas, the Guangdong–Hong Kong–Macao Greater Bay Area is entering the stage of service economy and innovation economy, and the demand for producer services is growing rapidly. The focus of regional cooperation within the Guangdong–Hong Kong–Macao Greater Bay Area has begun to shift to service trade, and the depth and breadth of cooperation has continued to increase. In conclusion, in the critical period of world economic recovery and China’s economic transformation and development, studying the international competitiveness and industrial synergy of service trade in the Guangdong–Hong Kong–Macao Greater Bay Area has strong practical significance.

At present, studies on the Guangdong–Hong Kong–Macao Greater Bay Area have mainly come from Chinese scholars. The relevant literature includes two categories: one is the study of the service trade in the Greater Bay Area, and the other is the study on the synergetic development of industries in the Greater Bay Area.

### 1.1. Studies on Service Trade in the Greater Bay Area

Scholarly studies are mainly divided into two aspects. One is the quantitative analysis of the liberalization and international competitiveness of service trade in Guangdong, Hong Kong, and Macao. Chen and Liu [2] used the service trade liberalization model and RCA index to analyze the degree of freedom and competitiveness of service trade between Guangdong, Hong Kong, and Macao. Zhang and Zhu [3] used the gravitational equation to evaluate the effect of CEPA on the service trade between Hong Kong and the Mainland. Chen and Chen [4] used MS, NTB, RCA, and other indexes to measure the international competitiveness of Macao’s service trade and analyzed the influencing factors by cointegration equations. Yang and Chen [5] analyzed the effects of service trade liberalization in Hong Kong, Macau, and the Mainland under CEPA by an extended gravity model. Xiao and Lin [6] compared the service trade competitiveness of the Guangdong–Hong Kong–Macao Greater Bay Area with other bay areas through IMS, TC, and RCA indexes as well as the competitiveness of different service industries in the Guangdong–Hong Kong–Macao Greater Bay Area. The second is to put forward the development countermeasures of service trade in Guangdong, Hong Kong, and Macao from the qualitative perspective. Liu and Zhu [7] proposed suggestions for accelerating the development of Shenzhen’s service trade based on the institutional arrangements for service trade. Chen and Liu [8] proposed suggestions for the development of service trade in Guangdong, Hong Kong, and Macao in terms of market access, mutual recognition of industry qualifications, and building a business environment. Xu [9] proposed the promotion of the development of Macao’s service trade by appropriate diversification of industries, training of professionals, and strengthening of cooperation between Macao and the Mainland.

### 1.2. Studies on the Industrial Synergetic Development of the Greater Bay Area

Studies in this field are concentrated after 2017, with comparatively few achievements that can mainly be divided into two categories. The first is to conduct qualitative research from the aspects of mechanism, mode, and path. Shen [10] proposed building a coordinated development system of urban agglomerations in the Greater Bay Area by elements, functions, organization, and connections. Xiang and Yang [11] put forward the industrial coordination development mechanism of the Guangdong–Hong Kong–Macao Greater Bay Area by the industrial division of labor, collaborative research and development, market expansion, and the cooperation model of manufacturing, service industry, and industrial integration. Wu [12] put forward the coordinated development path of cities in the Greater Bay Area from the aspects of industrial structure upgrade, coordination mechanism construction, and urban collaborative development. Based on the background of the new era, Fu and Liu [13] analyzed the basis, effectiveness, and path of the synergetic development of the Guangdong–Hong Kong–Macao Greater Bay Area. The second is to use various measurement methods to quantitatively analyze the degree of regional industrial synergy. Lin [14] constructed an evaluation system from the three dimensions of economic development, social development, and marketization system, and measured the regional synergy between Guangdong, Hong Kong, and Macao by the gray correlation model. Chen and Lin [15] used location entropy and the grey correlation model to measure the industrial synergy of 19 industries in the Guangdong–Hong Kong–Macao Greater Bay Area. Zhang et al. [16] used the composite system synergy model to measure the order and synergy of industrial development in the Guangdong–Hong Kong–Macao Greater Bay Area. Song et al. [17] analyzed the trade competition and cooperation between Guangdong, Hong Kong, and Macao with the help of principal component analysis, which found that the trade ties between the three cities continue to strengthen. However, it is still necessary to strengthen cooperation to give full play to their respective advantages and realize the development of trade dislocation. Wen et al. [18] used GIS network capture technology and modified gravity model, combined with railway traffic to measure the strength of economic spatial connection, and conducted research on the measurement of urban spatio-temporal evolution and coordinated development in the Guangdong–Hong Kong–Macao Greater Bay Area.

Through the literature review, it can be found that the current studies on service trade in a single area of the Guangdong–Hong Kong–Macao Greater Bay Area have produced many achievements, but there are few quantitative analyses and entire studies. Most of the studies on the coordinated development of industries in the Greater Bay Area have been analyzed from the perspective of regional city synergy, and the service trade is not regarded as the study object of the coordinated development of the Greater Bay Area from the industrial perspective. In light of this, based on the data from 2014 to 2018, this article analyzed the overall situation, the import and export structure, and the international competitiveness of various industries of service trade in the Guangdong–Hong Kong–Macao Greater Bay Area, and measures the level of coordination of regional service trade in the Greater Bay Area with the help of the gray correlation model and the entropy method. It is hoped that through these analyses, we will: (a) find the coordinated development direction of the various sub-sectors of the Greater Bay Area service trade; (b) establish a quantitative method to measure the level of synergy in the Greater Bay Area service trade and verify its rationality; and (c) propose relevant suggestions for the coordinated development of trade in services in the Greater Bay Area.

The main contributions of this paper are as follows. First, the research perspective is relatively new. The study on the competitiveness of service trade in the Guangdong–Hong Kong–Macao Greater Bay Area from industrial synergy proposes suggestions for the coordinated development of the Greater Bay Area service trade, which enriches the relevant results of quantitative research and overall research. Second, by referring to scholars’ measurement model of regional and industrial synergy degree, this paper proposes a regional industrial synergy degree method by comprehensively applying the entropy weight method and grey correlation analysis method, which verifies the rationality and applicability of the method. 

The rest of the paper is structured as follows. Section 2 assesses the international competitiveness of trade in services in the Guangdong–Hong Kong–Macao Greater Bay area. Section 3 uses the grey correlation model and the entropy weight method to quantitatively analyze the synergy level of the Greater Bay Area service trade. Finally, Section 4 presents our conclusions and suggestions.

## 2. Evaluation of International Competitiveness of Service Trade in the Guangdong–Hong Kong–Macao Greater Bay Area

To facilitate statistical analysis and objectively reflect the overall development level of service trade in the Guangdong–Hong Kong–Macao Greater Bay Area, this paper used Hong Kong, Macao, Guangzhou, and Shenzhen as samples for comparison. On one hand, the four cities are the four cities with the largest scale of service trade in Guangdong, Hong Kong, and Macao. The total service trade of Guangzhou and Shenzhen in 2018 accounted for four-fifths of the entire Guangdong area. On the other hand, the four cities have been identified by China as the core engines for the development of the Greater Bay Area, and their positioning and role are very important.

### 2.1. Overall Situation of Trade in Services in the Guangdong–Hong Kong–Macao Greater Bay Area

It can be seen from Table 1 that from 2014 to 2018, the total trade in services between Hong Kong and Macau declined in 2015 and 2016, and then rebounded in 2017. By 2018, Hong Kong reached a historical high of USD 195.838 billion and Macau reached USD 48.483 billion, which is close to the historical high of USD 48.959 billion in 2014. The total volume of service trade in Guangzhou has always maintained a stable development trend, with an average growth rate of 18.5%, which is much higher than the average growth rate of 4.9% in China and 2.7% worldwide. Due to the change in statistical caliber, the total volume of service trade in Shenzhen dropped sharply in 2017, but rebounded in the next year, with an increase of 23.9% that year, which was higher than the same period of 13.8% in China and 8.4% in the world.

It can be seen from Table 2, that from 2014 to 2018, the net export of the service trade of Hong Kong and Macao showed an overall growth trend and a long-term surplus. Guangzhou and Shenzhen showed a long-term deficit state, and the deficit increased year by year just like the overall situation in China.

After calculating and collating data from the WTO, the Guangdong Provincial Department of Commerce, and the Chinese Ministry of Commerce, it was found that in 2018, the service trade of Hong Kong, Macao, Guangzhou, and Shenzhen accounted for 14.1%, 69.8%, 24.5%, and 13.5% of their foreign trade, respectively, among which Macao and Guangzhou accounted for a comparatively high proportion. Compared with the world average level of 22.3% in the same period, Hong Kong and Shenzhen had a large gap. In general, the total proportion of the four cities was only 18.4%, which was lower than the world level in the same period. The development level of service trade in other cities in the Guangdong–Hong Kong–Macao Greater Bay Area lags more, but there will be huge growth momentum in the future, and there is a large space for the development of the service trade.

### 2.2. Structure of Service Trade in Guangdong-Hong Kong-Macao Greater Bay Area

According to the classification of “International Trade in Services Statistics Manual” and the characteristics of service trade in four cities, this paper selected eight major industries for analysis. As can be seen from Table 3, in 2018, transport, travel, and financial services played an important role in Hong Kong’s service exports, especially transport and travel services, which both accounted for about 30%, showing obvious advantages. Among Hong Kong’s service imports, travel, transport, and other commercial services accounted for a comparatively high proportion, all of which were more than 15%. Among Macao’s service exports, the travel service, mainly based on the gambling industry, accounts for 92.6% of its service exports, with an absolute advantage. However, the “domination in one industry” also has some risks, which limits the development of other industries. Among Macau’s service imports, travel, other commercial services, and financial services accounted for more than 10%. Among Guangzhou’s service exports, travel and other commercial services account for more than 30%, showing obvious advantages. Transport and computer information services accounted for more than 10%, where there are certain advantages. Among Guangzhou’s service imports, travel, transport, and computer information services account for more than 10%. Among Shenzhen’s service exports, other commercial services account for more than 30%, with the most obvious advantages. Transport, travel, and computer information services accounted for more than 15% of the advantages, much like the distribution of China. The service import situation of Shenzhen is similar to that of Guangzhou, the only difference being that the import of the intellectual property service also accounts for more than 10%.

From the import and export structure in the service trade of the four cities, the export of the service trade in the Greater Bay Area is dominated by traditional industries, and other emerging industries except that computer information services account for a comparatively small proportion, especially the intellectual property services, which are much lower than the proportion worldwide. The proportion of travel services in service imports is tremendous, and the four cities were all higher than the world average. The Greater Bay Area needs to gradually adjust and improve its service import and export structure. At the same time, the structural differences between different cities were also obvious. There is a large space for cooperation among different industrial sectors in the whole Greater Bay Area. Accelerating the coordinated development among different cities in the region is conducive to realizing internal resource complementarity and expanding and strengthening their respective competitive industries.

### 2.3. Evaluation of International Competitiveness of Service Trade in the Guangdong–Hong Kong–Macao Greater Bay Area

To uncover the competitive industries and collaborative development direction of different regions in the Greater Bay Area, this paper selected two commonly used trade performance evaluation indicators, TC and RCA, to measure the international competitiveness of different service trade industries in the four cities.

#### 2.3.1. Trade Competitiveness Index (TC)

The equation of the TC index is as follows:

TC = (*S_x_* − *S_j_*)/(*S_x_* + *S_j_*) (1)

Among them, *S_x_* represents the service export value of a certain region or industry and *S_j_* represents the service import value of a certain region or industry.

It can be seen from Table 4 that only the insurance industry in Hong Kong had an average TC value of less than zero, and the average TC value of other industries was greater than zero. Among them, the industry with strong competitive advantage was the financial industry (0.59), and the industries with weak competitive advantage was transport (0.27), travel (0.16), other commercial services (0.09), and computer information services (0.21). The industries where the average TC value of Macau was greater than zero included travel (0.93), transport (0.18), and financial services (0.28). In particular, the average TC value of the travel industry was very close to 1. The industries where the average TC value was less than zero included insurance (−0.33), computer information services, (−0.19), and other commercial services (−0.19). The industries where the average TC value in Guangzhou was greater than zero include construction (0.49), transport (0.05), and other commercial services (0.39). The industries where the average TC value of Shenzhen was greater than zero included construction (0.62), other commercial services (0.49), and computer information services (0.16). From the perspective of development trends, most industries in the four cities were in a stable development situation, and only the two traditional industries of travel and transport saw a significant decline.

According to the comparison in Table 5, Hong Kong and Macao have strong competitive advantages in financial services and Macao has strong competitive advantages in travel services, while these industries were the industries where Shenzhen and Guangzhou have large or extremely competitive disadvantages. In terms of Shenzhen and Guangzhou, construction and other commercial services have strong competitive advantages, while Macao and Hong Kong have weak competitive advantages in these two industries.

#### 2.3.2. Revealed Comparative Advantage Index (RCA)

The equation of RCA index is as follows:

(2) $$\mathrm{RCA}=(S_{i}÷S_{x})÷(W_{i}÷W_{x})$$

Among them, *S_i_* represents the export value of *i* service in a certain place; *S_x_* represents the total export value of its service trade; *W_i_* represents the global export value of *i* service; and *W_x_* represents the total export value of the global service trade.

From Table 6, we can see the development of the RCA index of each industry in the service trade in the four cities. In Hong Kong, the industries where the average RCA index value was higher than 1.25 included transport (1.59), travel (1.36), and financial (2.12), and other industries where the average RCA index value was less than 0.8. In Macau, the only industry with an average RCA index higher than 2.5 was travel (3.77), and the average RCA index for the remaining industries was all below 0.8. In Guangzhou, the industries where the average RCA index value was higher than 0.8 included travel (1.57), transport (0.98), computer information services (1.22), and other commercial services (1.20). In Shenzhen, the industries where the average RCA index value was higher than 0.8 included construction (2.89), computer information services (1.18), and other commercial services (1.33). It can be seen from the change trend in the RCA value that most of the industries in the four cities are developing well, and only the tourism industry is experiencing a comparatively obvious decline.

According to the comparison in Table 7, the travel service with strong competitiveness in Hong Kong and Macao was the industry with weak competitiveness in Shenzhen. Hong Kong’s highly competitive financial and transport services were precisely the industries in which Macao and Shenzhen were less competitive. Meanwhile, the construction, computer information services, and other commercial services with strong international competitiveness in Guangzhou and Shenzhen were the industries with weak international competitiveness in Hong Kong and Macao.

Through a comprehensive analysis of the overall situation of service trade in the four cities and the TC index and RCA index of each industry, there were obvious differences in the dominant industries of the four cities, the industry complementarity among them was very strong, and the whole Guangdong–Hong Kong–Macao Greater Bay Area has great potential for the coordinated development of service trade.

## 3. Empirical Analysis on the Synergy Degree of Service Trade in the Guangdong–Hong Kong–Macao Greater Bay Area

### 3.1. Analysis Framework

In order to further analyze the level of coordinated development of service trade in the Guangdong–Hong Kong–Macao Greater Bay Area, it is necessary to measure the degree of coordination among different regions. In recent years, research on synergy in different research fields has begun to increase. Scholars have used various measurement methods to analyze the synergy of the system quantitatively. The measurement methods mainly include data envelopment analysis [19], network analytic hierarchy process [20], coupling coordination degree model [21,22,23], composite system model [24,25,26,27], entropy and grey correlation model [28,29,30,31], SIM model [32], VAR model [33], FH model [34], distance coordination model [35,36], dispersion method and share method [37], system evolution method [38], etc. These measurement methods have been widely used in measuring the degree of synergy between different regions and industries and provide a theoretical basis for constructing the model in this article. This paper considered the collaborative development level of service trade in the Guangdong–Hong Kong–Macao Greater Bay Area as a complex system *M* = {*M*_1_, *M*_2_, *M*_3_, *M*_4_}. *M*_1_ is the Hong Kong service trade subsystem, *M*_2_ is the Macao service trade subsystem, and *M*_3_ is the Guangzhou service trade subsystem, and *M*_4_ is the Shenzhen service trade subsystem. The synergy of the composite system can be comprehensively evaluated by measuring the correlation between different subsystems. At the same time, because there are horizontal and vertical connections in the same industry among subsystems, the degree of correlation between subsystems can be measured by the degree of correlation between the RCA values of each department. Therefore, based on learning from the scholar’s above-mentioned model, this paper selected location entropy and gray correlation model for measurement. The model has no requirements on the sample size and distribution law, the calculation is relatively simple, and it has a wide range of adaptability. First, we used the gray correlation model to determine the correlation degree of the service trade industry among the various subsystems, and then calculated the overall synergy degree of the composite system of the Guangdong–Hong Kong–Macao Greater Bay Area through the entropy method.

### 3.2. Model Establishment

Step 1: Determine the reference sequence and the comparison sequence. Taking the RCA values of the eight industries in service trade in Hong Kong, Macao, Guangzhou, and Shenzhen from 2014 to 2018 as a reference sequence *X*_0_ = {*X*_0_(1), *X*_0_(2),..., *X*_0_(*n*)}, take another place. The RCA values of the eight industries in the service trade are used as the comparison sequence *X_i_* = {*X_i_*(1), *X_i_*(2),..., *X_i_*(*n*)}.

Step 2: Calculate the grey absolute correlation degree, see method shown below:

- Zero imaging of sequence starting point

(3) $$X_{0}^{0}(n)=X_{0}(n)-X_{0}(1),X_{{}_{i}}^{0}(n)=X_{i}(n)-X_{i}(1)$$

- Calculation |*S*_0_|,|*S_i_*|,|*S_i_*−*S*_0_|

(4) $$\begin{array}{l} \left|S_{0}\right|=\left|\sum_{k=2}^{n-1}X_{0}(k)+\frac{1}{2}X_{0}(n)\right|,\left|S_{i}\right|=\left|\sum_{k=2}^{n-1}X_{i}(k)+\frac{1}{2}X_{i}(n)\right|,\\ \left|S_{i}-S_{0}\right|=\left|\sum_{k=2}^{n-1}\left(X_{i}(k)-X_{0}(k))+\frac{1}{2}(X_{i}(n)-X_{0}(n)\right)\right| \end{array}$$

- Calculate the grey absolute correlation degree (*ɛ*_0*i*_)

(5) $$\varepsilon_{0i}=\frac{1+\left|S_{0}\right|+\left|S_{{}_{i}}\right|}{1+\left|S_{0}\right|+\left|S_{i}\right|+\left|S_{i}-S_{0}\right|}$$

Step 3: Calculate the gray relative correlation degree.

Suppose *X′*_0_ and *X′_i_* (*X’_i_* = *X_i_*/*X*_1_) are the initial value images of *X*_0_ and *X_i_*, respectively (none of the initial values are zero), then the absolute gray correlation degree of *X*′_0_ and *X*′*_i_* is called the gray relative degree of *X*_0_ and *X_i_*, represented by *r_0i_*.

Step 4: Calculate the grey comprehensive correlation degree.

(6) $$\rho_{0i}=\theta \varepsilon_{0i}+(1-\theta )r_{0i},\theta \in \left[0,1\right]$$

Step 5: Calculate the regional synergy degree of the industry.

This paper regarded the Guangdong–Hong Kong–Macao Greater Bay Area as a whole and used the comprehensive correlation degree of service trade between different cities in 2014–2018 as a reference series, used the entropy method to determine the contribution of different cities, and then conducted comprehensive calculations to obtain the Guangdong–Hong Kong–Macao Greater Bay Area. The calculation methods and steps are listed below:

- Determine the reference sequence (*Y_ij_*)

(7) $$Y_{ij}=\frac{\rho_{0i}-\min(\rho_{0i})}{\max(\rho_{0i})-\min(\rho_{0i})}$$

- Calculate the information entropy (*E_j_*)

(8) $$E_{j}=-\frac{1}{\ln m}\sum_{i=1}^{m}P_{ij}\ln P_{ij},P_{ij}=Y_{ij}/\sum_{i=1}^{m}Y_{ij}$$

- Calculate the contribution of each indicator (*W_j_*)

(9) $$W_{j}=\frac{1-E_{j}}{m-\sum_{j=1}^{m}E_{j}},\sum_{j=1}^{m}W_{j}=1$$

- Calculate the comprehensive synergy degree (*V_i_*)

(10) $$V_{i}=\sum_{i=1}^{n}W_{j}Y_{ij},V_{i}\in \left[0,1\right]$$

At the same time, the level of synergy was divided into five levels according to the value of the synergy degree, as shown in Table 8.

### 3.3. Data Calculation

According to Equations (3)–(6) and the RCA values of service trade subsectors in Hong Kong, Macao, Guangzhou, and Shenzhen in Table 6, take *θ* = 0.5 and group them in pairs to calculate the gray comprehensive correlation degree of Guangzhou–Shenzhen, Guangzhou–Hong Kong, Guangzhou–Macao, Shenzhen–Hong Kong, Shenzhen–Macao, and Hong Kong–Macao, respectively, and the calculation results are as the following Table 9:

According to Equations (7)–(9), the information entropy value and contribution degree of service trade correlation degree among different regions of the Guangdong–Hong Kong–Macao Greater Bay Area were calculated, and the calculation results are as following Table 10.

According to Equation (10), the comprehensive synergy degree of service trade in the Guangdong–Hong Kong–Macao Greater Bay Area is as following Table 11.

## 4. Conclusions and Suggestions

Based on the analysis above, the main conclusions are as follows:

(1) Through the analysis of the overall situation of service trade and the TC index and RCA index of each industry in Hong Kong, Macao, Guangzhou, and Shenzhen, the overall development trend of service trade in the Guangdong–Hong Kong–Macao Greater Bay Area is comparatively good, and the industrial competitiveness has been increasing year by year. There are obvious differences in the advantageous industries in the four cities, and the industries in different cities are highly complementary. The entire Guangdong–Hong Kong–Macao Greater Bay Area has great potential for the coordinated development of internal service trade.

(2) Judging from the grey comprehensive correlation degree of service trade among different cities in Hong Kong, Macao, Guangzhou, and Shenzhen, the degree of industrial correlation among all cities was greater than 0.6, indicating that the degree of industrial correlation between each other was comparatively high. Among them, the top three industrial correlation degrees were Guangzhou–Shenzhen, Guangzhou–Hong Kong, and Shenzhen–Hong Kong, which reached 0.8648, 0.7748, and 0.7179, respectively, indicating that the industrial synergy among Hong Kong, Guangzhou, and Shenzhen is good. The average industrial correlation degrees between Macau and Hong Kong, Guangzhou and Shenzhen were 0.6893, 0.6517, and 0.6041, respectively, which were all lower than 0.7, indicating that the industrial synergy between Macau and the three places is comparatively backward.

(3) From the comprehensive synergy degree of service trade in the Guangdong–Hong Kong–Macao Greater Bay Area, the overall trend is on the rise. Except for 2015, the remaining years were all above 0.5, and the overall synergy level is in the transition stage from the moderate to high synergy level.

Based on the above conclusions, from the perspective of the coordinated development of trade in services among the four regions, the following suggestions can be made to further enhance the international competitiveness of trade in services in the Greater Bay Area:

(1) Accelerate industrial integration and green transformation, and promote the coordinated development of regional trade in services. At present, the service trade in Guangdong, Hong Kong, and Macao is developing well, and the trade structure has been optimized to a certain extent. However, the level of development still lags far behind the three famous bay areas, mainly because the internal industrial structure is unreasonable. The distinctive industries and development potentials of Guangdong, Hong Kong, and Macao are quite different, and they are highly complementary to each other in the service trade. Guangdong should integrate Hong Kong and Macau’s international experience and professional advantages in finance, R&D and design, talent training, management consulting, shipping, tourism, etc., with the manufacturing system of the Pearl River Delta region through the service trade, thus promoting industrial integration, improving the development level and proportion of producer services, and speeding up the green transformation of the industry. At the same time, the best way for Hong Kong to consolidate its international status in finance, trade, and shipping industries, and for Macao to achieve appropriate industrial diversification is to accelerate its integration into the Greater Bay Area and develop the Guangdong and mainland markets through the service trade to expand the breadth and depth of its service industry and achieve sustainable development. In addition, it is necessary to focus on the goal of building an international shipping, logistics, trade, financial center as well as a national innovation center in the Greater Bay Area to scientifically integrate the superior industries of service trade in the four cities, establish a new type of competition and cooperation, and create a comprehensive and dislocation for a developed system of in-depth cooperation in the high-end service industry. While consolidating traditional industries, we should accelerate the development of emerging industries such as patent licensing fee services and computer information services to maximize the international competitiveness of the Greater Bay Area’s service trade.

(2) Establish a coordinated development management system to promote the liberalization of trade in services in the Greater Bay Area. In recent years, Guangdong, Hong Kong, and Macao have signed a series of agreements, and the degree of openness in the service trade has gradually increased. However, the mutual service trade has not formed a joint force, and all kinds of invisible trade barriers still exist. The fundamental reason is that the in-depth cooperation in the service trade in Guangdong, Hong Kong, and Macao is restricted by political systems, legal systems, tariff systems, and industry standards [39]. Therefore, it is necessary to strengthen the top-level design based on the principle of the “one country, two systems” policy, and establish a management system for the coordinated development of trade in services in the Greater Bay Area. Combining the planning of the Qianhai, Hengqin, and Nansha Free Trade Zones and the implementation of the CEPA Negative List, Guangdong can be the “first try” in the Guangdong–Hong Kong–Macao Greater Bay Area, granting larger pilot rights for the development of negative lists, and formulating simplified guidelines for Hong Kong and Macao, tailoring a “minimalist version” of a negative list for Hong Kong and Macau, and take the lead in liberalizing the requirements for investor qualifications, shareholding ratio restrictions and business scope in the service trade field, and create an upgraded version of CEPA. In addition, all regional governments should optimize the relevant laws and regulations under the framework of CEPA, introduce dispute settlement mechanisms in the field of service trade, expedite the removal of institutional and technical barriers, eliminate administrative and industry monopolies, accelerate the market connection between the Guangdong and Hong Kong and Macao service industries, and establish compliance. The Guangdong–Hong Kong–Macao actual and international standard service trade rule system will expand the opening and cooperation of service industries in Guangdong–Hong Kong–Macao and continue to promote the integration of the service trade in the Greater Bay Area [40].

(3) To improve the overall external service level of the region through the open markets and platforms in Hong Kong and Macao. In the Guangdong–Hong Kong–Macao Greater Bay Area, the development level of the service trade in the nine cities in Guangdong was significantly lower than that of Hong Kong and Macau. This uneven development in the region will affect the sustainable development of service trade in the Greater Bay Area. As a global trade and financial center, Hong Kong has a high degree of openness and close contacts with other countries. It has been selected by international institutions as the world’s freest economy for 25 years in a row. As one of the low-tax regions in the world, Macao has comparatively free flow of various resources and has close contacts with Portuguese-speaking countries. It has become a platform for business cooperation between China and Portuguese-speaking countries. Under the new situation, the Guangdong–Hong Kong–Macao Greater Bay Area should make full use of the open business environment and international platforms of Hong Kong and Macau, absorb advanced experience in foreign service industries, accelerate the construction of a new open economic system that conforms to international practices, expand the overall level of openness of the service industry, and promote high-quality development of the service industry. At the same time, it should guide companies in the Greater Bay Area to closely follow the “the Belt and Road” initiative, use Hong Kong and Macau as a springboard to invest in related infrastructure projects along the “the Belt and Road”, and give full play to the advantages of Guangdong’s construction services. Hong Kong and Macau provide these companies with professional services such as project financing and management, so that these companies can develop for a long time and realize the “group abroad” of the service industry of Guangdong, Hong Kong, and Macau [41].

Based on the data from 2014 to 2018, this paper analyzed the overall situation, import and export structure, and international competitiveness of various departments of service trade in the Guangdong–Hong Kong–Macao Greater Bay Area. It proposes a method for measuring the regional industrial synergy degree based on the entropy and gray correlation models. The measurement method is helpful to reveal the level and objective law of the coordinated development of service trade in the Guangdong–Hong Kong–Macao Greater Bay Area and guide the coordinated development of service trade in the Greater Bay Area. However, due to the complexity of regional industrial synergy and the impact of economic, technological, geographical, policy, and other factors, the quantitative research based on economic data in this study cannot measure the internal factors of the system comprehensively. In future work, we should use more data and quantitative policy information and try to introduce geoeconomics theory to explain the spatial pattern, evolutionary laws, and mechanisms of coordinated development in depth. In addition, due to the limitations of the data, this study was limited to the four regions of Guangzhou, Shenzhen, Hong Kong, and Macau, which reduced the regional differences to a certain extent. However, analysis of a broader area may provide more interesting results.

## Figures and Tables

**Table 1:** Comparison of the total service trade in four cities (unit: USD 100 million).

	2014	2015	2016	2017	2018
Hong Kong	1806.43	1781.90	1727.54	1811.17	1958.38
Macau	489.59	371.81	367.62	426.96	484.83
Guangzhou	243.60	291.70	378.10	457.50	480.95
Shenzhen	1006.10	1160.10	1069.30	571.70	708.45
China	6489.43	6505.45	6575.43	6905.22	7856.57
World	101,207.50	96,261.40	96,992.10	103,877.30	112,548.50

**Table 2:** Comparison of net export of service trade in four cities (unit: USD 100 million).

	2014	2015	2016	2017	2018
Hong Kong	330.27	303.36	241.20	260.85	320.00
Macau	414.87	295.85	291.06	338.62	386.87
Guangzhou	9.00	21.06	72.90	108.30	126.87
Shenzhen	132.50	95.10	216.90	126.54	180.01
China	2127.70	2154.05	2409.03	2377.44	2554.81

**Table 3:** Comparison of import and export structure in service trade of four cities in 2018 (unit: %).

	Hong Kong		Macao		Guangzhou		Shenzhen		China		World	
	Export	Import	Export	Import	Export	Import	Export	Import	Export	Import	Export	Import
Transport	28.78	22.61	1.60	9.60	18.27	10.51	17.23	11.47	15.96	20.80	17.62	22.15
Travel	32.24	32.59	92.60	28.70	36.61	49.78	19.37	38.20	14.89	53.18	24.90	25.60
Insurance	1.31	1.85	1.22	4.08	0.94	1.34	1.35	1.03	1.86	2.28	2.49	3.56
Financial	20.83	7.39	2.49	14.42	0.06	0.39	0.23	0.91	1.31	0.41	8.49	4.61
Construction	0.12	0.16	0.00	0.82	1.25	0.25	6.80	1.48	10.03	1.65	1.89	1.78
Computer Information Services	2.73	2.49	0.10	1.89	11.73	17.73	17.33	13.41	17.75	4.57	10.50	6.36
Intellectual Property	0.69	2.49	NA	NA	0.76	7.37	2.61	14.12	2.11	6.84	6.99	7.95
Other Commercial Services	13.81	15.23	2.08	22.76	30.49	6.78	32.26	8.14	26.37	9.08	21.93	22.40

**Table 4:** Development of TC index in sub-sectors of service trade in the four regions.

Departments and Cities		2014	2015	2016	2017	2018	Average	Trend
Transport	Hong Kong	0.27	0.26	0.25	0.27	0.28	0.27	→
	Macau	0.11	0.10	0.16	0.15	0.20	0.18	↑
	Guangzhou	0.15	0.06	0.02	0.03	0.01	0.05	↓
	Shenzhen	0.01	0.04	0.07	0.06	0.06	0.04	→
Travel	Hong Kong	0.27	0.22	0.15	0.14	0.16	0.19	↓
	Macau	0.94	0.92	0.92	0.94	0.93	0.93	→
	Guangzhou	0.16	0.22	0.32	0.38	0.40	0.30	↓
	Shenzhen	0.31	0.29	0.42	0.46	0.54	0.40	↓
Insurance	Hong Kong	0.09	0.05	0.01	0.01	0.01	0.03	→
	Macau	0.30	0.02	0.46	0.44	NA	0.16	↑
	Guangzhou	0.54	0.55	0.74	0.34	0.42	0.52	↑
	Shenzhen	0.10	0.03	0.01	0.14	0.12	0.03	↓
Financial	Hong Kong	0.60	0.60	0.58	0.58	0.60	0.59	→
	Macau	0.51	0.43	0.24	0.20	0.33	0.34	→
	Guangzhou	0.93	0.93	0.98	0.47	0.84	0.83	→
	Shenzhen	0.17	0.56	0.69	0.76	0.73	0.58	→
Construction	Hong Kong	0.02	0.01	0.01	0.02	0.02	0.01	→
	Macau	NA	NA	NA	NA	NA	NA	NA
	Guangzhou	0.54	0.63	0.09	0.70	0.48	0.49	→
	Shenzhen	0.70	0.64	0.64	0.67	0.46	0.62	→
Intellectual Property	Hong Kong	0.51	0.49	0.47	0.46	0.47	0.48	→
	Macau	NA	NA	NA	NA	NA	NA	NA
	Guangzhou	0.92	0.93	0.93	0.93	0.92	0.93	→
	Shenzhen	0.82	0.81	0.77	0.75	0.80	0.79	→
Computer and information services	Hong Kong	0.19	0.20	0.19	0.19	0.18	0.19	→
	Macau	0.29	0.32	0.42	0.50	0.51	0.41	→
	Guangzhou	0.09	0.02	0.41	0.54	0.44	0.47	→
	Shenzhen	0.65	0.38	0.16	0.27	0.13	0.16	→
Other Commercial Services	Hong Kong	0.08	0.08	0.08	0.10	0.09	0.09	→
	Macau	0.26	0.27	0.28	0.07	0.21	0.22	→
	Guangzhou	0.41	0.35	0.30	0.47	0.43	0.39	↑
	Shenzhen	0.55	0.54	0.52	0.44	0.40	0.49	→

Trend for samples (→:flat, ↑:rising, and↓:falling)

**Table 5:** Comparison results of the mean value of TC index in the service trade subsectors of the four cities.

Value	Implication	Hong Kong	Macau	Guangzhou	Shenzhen
TC > 0.6	Very Strong Competitive Advantage		Travel		Construction
0.3 < TC < 0.6	Strong Competitive Advantage	Financial	Financial	Construction and other Commercial Services	Other Commercial Services
0 < TC < 0.3	Slight Competitive Advantage	Transport, Travel, other Commercial Services, Computer Information Services	Transport and insurance	Transport	Computer Information Services
−0.3 < TC < 0	Slight Competitive Disadvantage	Construction, Insurance	Other Commercial Services		Transport and Insurance
−0.6 < TC < −0.3	Strong Competitive Disadvantage	Intellectual Property	Computer Information Services	Travel, Insurance, Computer Information Services	Travel and Financial
TC < −0.6	Very Strong Competitive Disadvantage			Financial, Intellectual Property	Intellectual Property

**Table 6:** RCA index development of service trade sub-sectors in the four cities.

Departments and Cities		2014	2015	2016	2017	2018	Average	Trend
Transport	Hong Kong	1.54	1.54	1.62	1.64	1.63	1.59	↑
	Macau	0.07	0.09	0.10	0.09	0.09	0.09	→
	Guangzhou	1.00	0.90	0.95	1.01	1.04	0.98	→
	Shenzhen	0.56	0.50	0.60	1.02	0.98	0.73	↑
Travel	Hong Kong	1.47	1.41	1.34	1.30	1.29	1.36	→
	Macau	3.86	3.78	3.72	3.78	3.72	3.77	↓
	Guangzhou	1.77	1.48	1.65	1.46	1.47	1.57	↓
	Shenzhen	0.43	0.38	0.45	0.90	0.78	0.59	→
Insurance	Hong Kong	0.42	0.49	0.55	0.54	0.52	0.51	→
	Macau	0.08	0.20	0.50	0.49	NA	0.32	→
	Guangzhou	0.19	0.22	0.23	0.32	0.38	0.27	↑
	Shenzhen	0.42	0.35	0.44	0.55	0.54	0.46	↓
Financial	Hong Kong	1.85	2.02	2.03	2.24	2.45	2.12	↑
	Macau	0.27	0.36	0.32	0.28	0.30	0.31	→
	Guangzhou	0.01	0.01	0.00	0.01	0.01	0.01	→
	Shenzhen	0.02	0.02	0.02	0.03	0.03	0.02	→
Construction	Hong Kong	0.16	0.08	0.07	0.06	0.07	0.09	→
	Macau	NA	NA	NA	NA	NA	NA	NA
	Guangzhou	0.44	0.47	0.29	0.64	0.66	0.50	↑
	Shenzhen	2.15	2.00	2.86	3.84	3.60	2.89	↑
Intellectual Property	Hong Kong	0.09	0.09	0.10	0.10	0.09	0.09	→
	Macau	NA	NA	NA	NA	NA	NA	NA
	Guangzhou	0.07	0.06	0.07	0.06	0.07	0.07	→
	Shenzhen	0.06	0.09	0.23	0.36	0.37	0.22	↑
Computer information services	Hong Kong	0.29	0.28	0.29	0.27	0.27	0.28	→
	Macau	0.01	0.02	0.01	0.01	0.01	0.01	→
	Guangzhou	1.37	1.24	1.34	1.03	1.12	1.22	↓
	Shenzhen	1.11	0.83	0.93	1.40	1.65	1.18	↑
Other Commercial Services	Hong Kong	0.56	0.59	0.61	0.61	0.60	0.60	→
	Macau	0.06	0.06	0.06	0.09	0.08	0.07	→
	Guangzhou	1.12	1.02	1.09	1.35	1.40	1.20	↑
	Shenzhen	1.23	1.10	1.26	1.58	1.47	1.33	↑

Trend for samples (→:flat, ↑:rising, and↓:falling)

**Table 7:** Comparison results of the average value of the RCA index in service trade sub-sectors of the four regions.

Value	Implication	Hong Kong	Macau	Guangzhou	Shenzhen
RCA > 2.5	Very strong competitiveness		Travel		Construction
1.25 < RCA < 2.5	Strong competitiveness	Transport, Travel, Financial		Travel	Other Commercial Services,
0.8 < RCA < 1.25	Slight competitiveness			Transport, Computer Information Services, other Commercial services	Computer Information Services
RCA < 0.8	Weak competitiveness	Insurance, construction, Computer Information services, Intellectual Property, Other Commercial Services	Financial, transport, Insurance, Computer Information Services, Intellectual Property, Other Commercial Services	Financial, Insurance, Intellectual Property	Transport, Travel, Insurance, Financial, Intellectual Property

**Table 8:** Regional synergy level division standard.

Synergy Value	0.8 ≤ *V_i_* ≤ 1	0.6 ≤ *V_i_* < 0.8	0.4 ≤ *V_i_* < 0.6	0.2 ≤ *V_i_* < 0.4	0 ≤ *V_i_* < 0.2
Synergy Level	Very High	High	Moderate	Low	Very Low

**Table 9:** Gray comprehensive correlation degree of service trade between different regions.

	Guangzhou–Shenzhen	Guangzhou–Hong Kong	Guangzhou–Macao	Shenzhen–Hong Kong	Shenzhen–Macao	Hong Kong–Macao
2014	0.9089	0.7665	0.6500	0.7321	0.6020	0.7108
2015	0.8823	0.7652	0.6399	0.7123	0.5894	0.5894
2016	0.9168	0.7601	0.6457	0.7805	0.6293	0.7144
2017	0.7920	0.7919	0.6626	0.6704	0.5951	0.7134
2018	0.8238	0.7904	0.6603	0.6942	0.6047	0.7185
Average	0.8648	0.7748	0.6517	0.7179	0.6041	0.6893
Sorting	1	2	5	3	6	4

**Table 10:** Information entropy value and contribution degree of correlation degree of service trade between different regions.

	Guangzhou–Shenzhen	Guangzhou–Hong Kong	Guangzhou–Macao	Shenzhen–Hong Kong	Shenzhen–Macao	Hong Kong–Macao
Information Entropy	0.8020	0.6992	0.7859	0.7724	0.7860	0.8612
Correlation Degree	0.1531	0.2326	0.1655	0.1760	0.1654	0.1073

**Table 11:** Comprehensive synergy degree of service trade in the Guangdong–Hong Kong–Macao Greater Bay Area.

Year	2014	2015	2016	2017	2018
Synergy Value	0.5157	0.2151	0.6408	0.5249	0.6182
Synergy Level	Moderate	Low	High	Moderate	High

## Data Availability

The data presented in this study are available in the WTO database at https://www.wto.org (accessed on 24 June 2021) and the Guangdong Provincial Department of Commerce website http://com.gd.gov.cn (accessed on 24 June 2021).

## References

[1] Lin L.L., Wu Z.C. (2020). The Enlightenment of German technological innovation experience to the Construction of Guangdong-Hong Kong-Macao Greater Bay Area as an international technological innovation Center. Sci. Technol. Manag. Res..

[2] Chen E., Liu J. (2013). Research on the path of service trade liberalization between Guangdong, Hong Kong and Macao. South. Econ..

[3] Zhang Y.W., Zhu T.Y. (2015). Does CEPA Promote Service Trade between Hong Kong and Mainland China. Int. Econ. Trade Explor..

[4] Chen E., Chen Z.Z. (2015). An empirical analysis on the international competitiveness of Macao’s trade in services and its influencing factors. J. Harbin Univ. Commer. (Soc. Sci. Ed.).

[5] Yang J., Chen E. (2018). The Economic Effects of Service Trade Liberalization under CEPA. Asia Pac. Econ..

[6] Xiao K.X., Lin L.M. (2019). A Comparative Study of the Competitiveness of Service Trade among the Guangdong-Hong Kong-Macao Greater Bay Area and other famous Bay Areas. City Watch.

[7] Liu W.L., Zhu K. (2015). Policy Suggestions on Accelerating the Development of Trade in Services in Shenzhen. Open. Rev..

[8] Chen H., Liu Y. (2018). Trade liberalization in services between Guangdong, Hong Kong and Macao: Theory, policy and practice. China Dev..

[9] Xu X.H. (2019). Research on the development trend and countermeasures of Macao service trade. North. Econ. Trade.

[10] Shen M.H. (2017). Construction Path of Collaborative Development of Urban Agglomeration in Guangdong-Hong Kong-Macao Greater Bay Area. Chin. J. Soc. Sci..

[11] Xiang X.M., Yany J. (2018). The mechanism and mode of industrial collaborative development in the Guangdong-Hong Kong-Macao Greater Bay Area. J. South China Norm. Univ. (Soc. Sci. Ed.).

[12] Wu W.X. (2019). Discussion on collaborative development path of urban agglomeration in Guangdong-Hong Kong-Macao Greater Bay Area. Jianghuai Forum.

[13] Fu Z.P., Liu J.L. (2021). Research on the Coordinated Development of Guangdong-Hong Kong-Macao Greater Bay Area in the New Era. Reg. Econ. Rev..

[14] Lin C.H. (2018). Evaluation of Hong Kong and Macao Coordinated Development Level under the Strategic Background of "Greater Bay Area" and Countermeasures Enlightenment. Asia Pac. Econ..

[15] Chen Y., Lin Z.H. (2018). A study on industrial cooperation between cities in the Guangdong-Hong Kong-Macao Greater Bay Area. J. Guangdong Univ. Financ. Econ..

[16] Zhang Y., Jian L.X., Mi S.J. (2019). The collaborative development of industries in the Guangdong-Hong Kong-Macao Greater Bay Area. J. Dalian Marit. Univ..

[17] Song Z.Y., Zhu Q.L., Xu J.Y. (2020). The relationship between trade competition and cooperation in the Guangdong-Hong Kong-Macao Greater Bay Area and its optimization path. Geogr. Res..

[18] Wen X., Gao W.X., Zhu J.X. (2021). Study on the measurement and coordinated development of urban spatiotemporal evolution in the Guangdong-Hong Kong-Macao Greater Bay Area. Stat. Decis..

[19] Shi M.K., Yu L.Y. (2020). Cooperative efficiency measurement of China’s provincial science financial system based on network data envelopment analysis and its time-spatial differences. J. Shanghai Univ. (Nat. Sci. Ed.).

[20] Li W., Xi Y.Q. (2017). The measurement of synergy of strategic emerging industry innovation System. Stat. Decis..

[21] Yang H. (2020). Research on infrastructure integration of 13 cities in Beijing-Tianjin-Hebei region: Based on coupled coordination degree model. Econ. Manag..

[22] Ning C.S., Li S.D. (2020). Dynamic evaluation of coordination degree between ecological protection and economic development in the Yellow River Basin. People’s Yellow River.

[23] Hua X.Y., Jin X.R., Lv H.P., Ye Y.F., Shao Y.H. (2021). The evolution and influencing factors of the spatiotemporal pattern of high-quality development coupling coordination: A case study of the counties in Zhejiang Province. Geogr. Sci..

[24] Brem A., Nylund P.A., Schuster G. (2016). Innovation and de facto standardization: The influence of dominant design on innovative performance, radical innovation, and process innovation. Technovation.

[25] Wang Y.D., Zhang B., Wu C., Xu Y.L. (2019). Research on the synergy measurement of high-tech industry innovation chain and capital chain: Based on the synergy model of composite system. Sci. Technol. Prog. Policy.

[26] Ma X. (2019). Evaluation of Beijing-Tianjin-Hebei regional economic synergy based on the model of composite system synergy. Ind. Technol. Econ..

[27] Zheng Y.W., Xue W.X. (2019). The driving forces of regional synergistic development in the Silk Road Economic Belt: Empirical study by stages based on Haken model. China Soft Sci..

[28] Song Y.Q., Li H.J., Wang Q., Li G.J. (2020). System synergy measurement method and empirical study based on Kolmogorov entropy. Oper. Res. Manag..

[29] Liu Y., Zhou L.Y., Geng C. (2017). Evaluation of the coordinated development of industries in Beijing-Tianjin-Hebei: Analysis based on location entropy and grey correlation. J. Cent. Univ. Financ. Econ..

[30] Du Y., Li S.L. (2021). The measurement of regional scientific and technological innovation ability based on the evaluation of the synergy of subsystems: Taking Gansu province as an example. Forum Sci. Technol. China.

[31] Ma C.P. (2018). Dissipative structure theory in the coordinated development of regional economy. J. Interdiscip. Math..

[32] Si L.B., Meng W.D. (2017). Evaluation of the synergy degree of the equipment manufacturing technology collaborative innovation mechanism: An empirical analysis based on the SIM model. Technoecon. Manag. Res..

[33] Li J.Q., Liu H.N. (2016). Possibility analysis of the upgrade and integration of the Beijing-Tianjin-Hebei industrial structure. J. Natl. Sch. Adm..

[34] He S.F., Yan H. (2018). Measurement and analysis of the level of regional financial coordinated development based on the F-H model: Taking Chengdu Plain Economic Zone as an example. J. Sichuan Adm. Inst..

[35] Kang H.C., Guo Z.X. (2019). Study on the coordinated development of regional logistics and regional economy based on the distance synergy model. J. Hebei Agric. Univ. (Soc. Sci. Ed.).

[36] Li J., Fan C.G., Yuan Q.M. (2017). Beijing-Tianjin-Hebei coordinated development level measurement based on distance coordination model. Sci. Technol. Manag. Res..

[37] Cao X.H., Li Y., Xu Y.H. (2017). The construction and comparative study of the measurement system of cross-strait economic integration. J. Shanxi Univ. Financ. Econ..

[38] Qiu H.Q., Luo J. (2019). Research on the measurement of regional coordinated development and spatio-temporal evolution under the new development concept: Taking the cooperative development area of southwest Fujian as an example. East China Econ. Manag..

[39] Mao Y.H., Yang S.W. (2019). Theoretical basis and institutional innovation of the construction of the Guangdong-Hong Kong-Macao Greater Bay Area. J. Sun Yat-Sen Univ. (Soc. Sci. Ed.).

[40] Ni W., Zhou S.H., Wei Z.Y. (2020). Research on the Development of Economic Integration in the Greater Bay Area—Based on the Analysis of the Guangdong-Hong Kong-Macao Greater Bay Area. Shanghai Econ. Res..

[41] Xu X.H. (2020). The international competitiveness of China’s wine industry: A case study from the perspective of trade performance evaluation. Price Theory Pract..

